# Co-digestion of microalgae with potato processing waste and glycerol: effect of glycerol addition on methane production and the microbial community†

**DOI:** 10.1039/d0ra07840a

**Published:** 2020-10-09

**Authors:** Yanghanzi Zhang, Gary S. Caldwell, Philip T. Blythe, Andrew M. Zealand, Shuo Li, Simon Edwards, Jin Xing, Paul Goodman, Paul Whitworth, Paul J. Sallis

**Affiliations:** School of Engineering, Newcastle University Cassie Building, Claremont Road Newcastle upon Tyne NE1 7RU UK yanghanzi.zhang@newcastle.ac.uk; School of Natural and Environmental Sciences, Newcastle University Ridley Building, Claremont Road Newcastle upon Tyne NE1 7RU UK; Department of Applied Sciences, Faculty of Health and Life Sciences, Northumbria University Newcastle upon Tyne NE1 8ST UK

## Abstract

The production of methane-rich biogas from the anaerobic digestion (AD) of microalgae is limited by an unfavorable biomass carbon-to-nitrogen (C/N) ratio; however, this may be ameliorated using a co-digestion strategy with carbon-rich feedstocks. For reliable plant operation, and to improve the economics of the process, secure co-feedstock supply (ideally as a waste-stream) is important. To this end, this study investigated the feasibility of co-digesting microalgae (*Chlorella vulgaris*) with potato processing waste (potato discarded parts, PPW_dp_; potato peel, PPW_p_) and glycerol, while monitoring the response of the methanogenic community. In this semi-continuous study, glycerol (1 and 2% v/v) added to mixtures of *C. vulgaris* : PPW_dp_ enhanced the specific methane yields the most, by 53–128%, whilst co-digestion with mixtures of *C. vulgaris* : PPW_p_ enhanced the methane yields by 62–74%. The microbial communities diverged markedly over operational time, and to a lesser extent in response to glycerol addition. The acetoclast *Methanosaeta* was abundant in all treatments but was replaced by *Methanosarcina* in the potato peel with glycerol treatment due to volatile fatty acid (VFA) accumulation. Our findings demonstrate that the performance of microalgae co-digestion is substantially improved by the addition of glycerol as an additional co-feedstock. This should improve the economic case for anaerobically digesting microalgae as part of wastewater treatment processes and/or the terminal step of a microalgae biorefinery.

## Abstract 2

Glycerol as an additional co-substrate enhanced methane yields by up to 128% when co-digestion with microalgae and potato waste.

## Graphical abstract

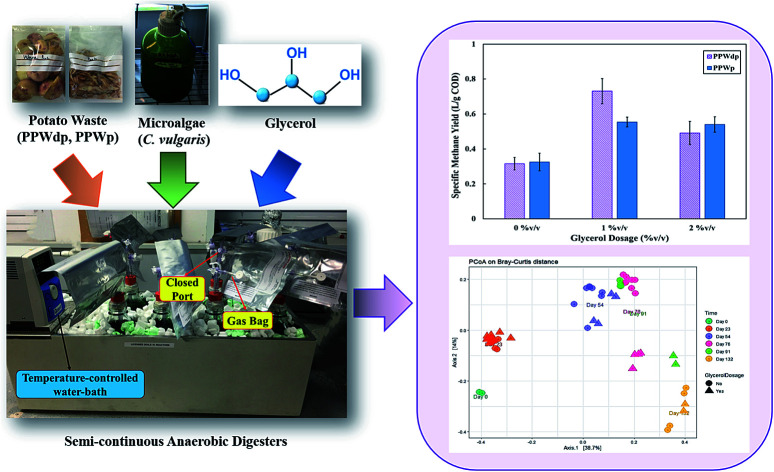

## 1. Introduction

In 2017, the UK government announced the Industrial Strategy, which set out four Grand Challenges. The Clean Growth Grand Challenge seeks to use low carbon technologies and efficient new materials to encourage the rapid growth of a clean economy. The development of new markets in areas such as smart energy systems and the ‘bio-economy’ are encouraged to accelerate progress to achieve this identified challenge.^1^ The ‘bio-economy’ refers to the use of renewable biological resources from land and sea to produce food, materials and energy. The production of renewable energy from biomass resources is one of the important options for sustainable development. Biomass energy in the form of heat, electricity and liquid fuels (biofuels) can be produced from a wide range of biomass sources such as virgin wood, energy crops, agricultural residues and food and industrial waste streams *via* various conversion processes.^2^ Microalgae have a number of advantages over conventional biomass, including high productivity, lower lignin content, less land requirement and high photosynthetic efficiency.^3,4^ There is growing interest in the mass production of microalgae for a range of biotechnology and bioengineering applications, ranging from the extraction of individual chemicals to the use of the entire biomass, often for fuels or environmental remediation activities.^5^ However, growing microalgae specifically for low value, high volume products such as biodiesel is well documented as being economically unfeasible using current culture and processing practices.^6^ Alternative approaches to valorising microalgae biomass have concentrated on developing more diverse business models, with the biomass contributing multiple income streams – commonly referred to as a biorefinery concept.^3,7^ A simpler scenario could involve microalgae contributing an ecosystem service during growth, *e.g.* remediating excess nutrients in a wastewater treatment process, with the resulting biomass processed for additional products.^8,9^ Anaerobic digestion (AD) is one such downstream process; indeed, in addition to AD improving the overall energy balance of wastewater treatment processes.^10^ AD is argued as a vital step in improving the economic case for microalgae biodiesel production.^11^

AD is a robust biochemical conversion process whereby macromolecules (carbohydrates, proteins and lipids) can be degraded by anaerobic microorganisms to generate methane-rich biogas, with the digestate potentially used as a nutrient fertilizer in agriculture.^12^ However, microalgae biomass is rich in protein which may contribute to an unbalanced carbon-to-nitrogen (C/N) ratio (typically within a range of 4.65 to 17), which is lower than the optimum range of 20 to 30 for AD.^13–16^ This imbalance may destabilise the AD process and reduce methane production, particularly if microalgae are used as a mono-digestion feedstock. Anaerobic co-digestion of two or more complementary feedstocks may cause a synergistic effect on their biodegradability, enhancing methane production.^17^ Microalgae have been reported to co-digest effectively with carbon-rich feedstocks such as waste paper and maize, thereby rebalancing the C/N ratio and increasing methane production.^14,18,19^ To further support the economic case for digesting microalgae, any carbon-rich feedstocks should come as waste-streams rather than be produced specifically for bioenergy production. Potato processing waste (PPW) is a main by-product of industrial potato processing such as manufacturing French fries, canned foods and starch products. PPW is rich in soluble organic matter, and its C/N ratio ranges from 12.1 to 30.0.^20,21^ Therefore, co-digestion with PPW is another option to improve methane production from microalgae. Previously, we demonstrated stable co-digestion of a freshwater microalga (*Chlorella vulgaris*) and a marine microalga (*Tisochrysis lutea*) with PPW in batch and semi-continuous modes.^4,22^ However, reliance on a single waste-stream could pose some operational risks to an AD plant,^23^ furthermore in order to improve the economics of the process, secure co-feedstock supply is important. Therefore, in the current study we introduce an additional level of feed complexity by including glycerol as a secondary co-digestion feedstock.

Glycerol is a main by-product of biodiesel production representing 10% w/w of the total product stream.^24–26^ By 2028, total global biodiesel production is predicted to reach 44 million tonnes, generating 4.4 million tonnes of glycerol.^27^ The rapid growth of the biodiesel industry has led to overproduction of crude glycerol and glycerol disposal is associated with environmental concerns.^28^ An alternative to disposal, which concomitantly mitigates its surplus production, is to convert the glycerol to other valuable products. Glycerol contains high concentrations of chemical oxygen demand (COD) and is easily degraded by acidogenic bacteria to produce organic acids.^29–31^ Therefore, glycerol is a potential feedstock for anaerobic co-digestion with low carbon feedstocks such as pig manure and, mixtures of strawberry and fish waste.^32–34^ Glycerol was also introduced to co-digest with algal biomass to boost methane production. For instance, Oliveira *et al.* (2015) investigated that co-digestion of macroalgae *Sargassum* sp. with glycerol and waste frying oil to improve methane production in batch BMP test.^35^ Their results showed that the addition of glycerol to these two feedstocks increased methane yields by 56%. Neumann *et al.* (2015) investigated that anaerobic co-digestion of 90% lipid-spent microalgae *Botryococcus braunii* with 10% glycerol in batch BMP tests, it was found that methane yields slightly increased compared to digestion with mono-substrate.^36^ However, the methane production rates were not significantly enhanced. AD of whole microalgae seems to be an optimum strategy in terms of energy balance if the microalgal cell-lipid composition is less than 40%.^11^ Therefore, glycerol is also considered to be a potential feedstock for microalgae co-digestion although there is little information available about co-digestion of whole microalgae with glycerol. Moreover, in order to avoid some potential AD plant's operational risks that caused by a single waste-stream co-substrate, the effect of microalgae co-digestion with PPW and glycerol on methane production needs to be evaluated.

Although many studies have reported the co-digestion of PPW or glycerol with other feedstocks, information on anaerobic co-digestion of microalgae with PPW and glycerol is limited; this is an important knowledge gap within the context of microalgae biorefineries given the likely incidental increase in glycerol production from transesterification as biodiesel from microalgae becomes a commercial reality.^7,37^ In addition, previous microalgae co-digestion studies mostly focused on evaluating the effects of environmental factors on the stability of the co-digestion process,^19^ few have assessed the impact on the microbial community. The current study aimed to enhance methane production from microalgae by co-digestion with PPW and glycerol. For semi-continuous co-digestion studies, the effect of glycerol dosage on methane production, process stability and microbial diversity/structural dynamics was investigated.

## 2. Methods and materials

### 2.1 Microalgae and co-substrates

*Chlorella vulgaris* strain (CCAP 211/63) was used in this study, and detailed information of cultivation and harvesting of microalgae were described by previous studies.^22,38^ Two categories of PPW were prepared; discarded parts (PPW_dp_) and peel (PPW_p_). The detailed information of preparation of two PPW feedstocks (PPW_dp_ and PPW_p_) were described by previous studies.^4,22^ The feedstocks of *C. vulgaris*, PPW_dp_ and PPW_p_ were characterised for their total solids (TS), volatile solids (VS), total chemical oxygen demand (COD_t_), and carbohydrate and protein content as well as C and N content.

The anaerobic seed inoculum was collected from a manure-based farm anaerobic digester located at the University owned Cockle Park Farm, Northumberland, UK. The characteristics of *Chlorella vulgaris*, PPW and seed inoculum are summarized in Table 1.

**Table 1:** Feedstock and inoculum characteristics

	*C. vulgaris*	PPW_dp_^a^	PPW_p_^b^	Inoculum
TS (g L^−1^)	2.7 ± 0.2^c^	16.0 ± 0.4	15.7 ± 0.1	18.6 ± 1.2
VS (g L^−1^)	2.4 ± 0.1	14.8 ± 0.4	13.9 ± 0.1	10.4 ± 0.9
VS/TS (%)	88.9 ± 3.2	92.2 ± 0.1	88.7 ± 0.1	56.1 ± 0.9
COD_t_ (g L^−1^)	3.5 ± 0.3	13.8 ± 0.2	12.5 ± 0.1	n.a.^d^
Proteins (% VS)	37.6 ± 4.0	13.0 ± 0.2	13.7 ± 1.1	n.a.
Carbohydrates (% VS)	23.8 ± 3.3	74.8 ± 0.1	69.0 ± 3.7	n.a.
C/N	6.4	40.8	28.6	n.a.

a PPW_dp_: potato discarded parts.

b PPW_p_: potato peel.

c Mean ± SD, *n* = 2.

d n.a.: not analysed.

A glycerol solution (Sigma-Aldrich, 4978, UK) with a purity of 86–89% was used as a co-substrate. The glycerol solution had a COD_t_ of 1888.0 ± 2.8 g L^−1^ and density of 1.25 kg L^−1^.

### 2.2 Operation of semi-continuous anaerobic digesters

Eight identical one litre Duran bottles (VWR, UK) with a working volume of 0.8 L were used as the semi-continuous co-digestion digesters, and detailed information of the digester configuration was described by Zhang *et al.* (2019).^4^ At the beginning of the experiment, all digesters were filled with 0.8 L of anaerobic inoculum, and flushed with N_2_ to ensure anaerobic conditions. The semi-continuous digesters were kept at constant temperature of 37 °C by a temperature-controlled water-bath. The digester was mixed by hand mixing before and after feeding.

The semi-continuous digesters were studied at four feeding conditions: the digesters were fed with mixtures of *C. vulgaris* : PPW_dp_ and glycerol (C1); mixtures of *C. vulgaris* : PPW_dp_ without glycerol (C2); mixtures of *C. vulgaris* : PPW_p_ and glycerol (C3); and mixtures of *C. vulgaris* : PPW_p_ without glycerol (C4). All digesters were fed every day during period I and every two days for the rest of periods. The hydraulic retention time (HRT) was set at 20 days with an overall operation time of 76–132 days. Detailed information of variation of organic loading rates (OLRs) and feedstock composition over the co-digestion process is summarized in Table 2. During period I, all digesters were fed with 100% PPW_dp_ or 100% PPW_p_. In period II, all digesters were fed a mixture of 25% *C. vulgaris* and 75% PPW_dp_ or 75% PPW_p_ based on the proportion of VS. A 25 : 75 ratio of *C. vulgaris* and PPW had the potential to provide an optimum C/N ratio and produce high methane yields compared to other tested mixing ratios (*e.g.* 75 : 25 and 50 : 50) as discussed by Zhang *et al.* (2019).^22^ Digesters C1 and C3 were also fed with glycerol, and the dosage of glycerol in the mixture was progressively increased from 1 to 2% v/v over period II. The glycerol dosages of 1 and 2% v/v were selected based on previous studies on anaerobic co-digestion of glycerol with other low carbon feedstocks.^29,30^ Periods III and IV are the experimental phases for digesters C1 and C2, where digester C1 was supplemented with different glycerol dosages, *i.e.*, 2 and 1% v/v for periods III and IV, respectively. Digester C2 was used as control digester, and fed with 25 : 75 *C. vulgaris* : PPW_dp_ without glycerol supplementation. Overall, digesters C1 and C2 were operated 76 days. For digesters C3 and C4, the operating phases started from periods III to VI. During periods III and IV, digester C3 was supplemented with 2% v/v of glycerol, but had no glycerol supplementation in period V. Glycerol was back to add to digester C3 at 1% v/v during period VI. Control digester C4 was only fed with 25 : 75 *C. vulgaris* : PPW_p_. Overall, digesters C3 and C4 were operated for 132 days.

**Table 2:** Organic loading rate (OLR) and feedstock composition for co-digesting *C. vulgaris* and potato processing waste (PPW) with or without glycerol

Feeding regime	Period	Operation time (days)	OLR (g COD per L per d)	Feed composition^a^		
				*C. vulgaris* (% VS)	PPW^b^ (% VS)	Glycerol (% v/v)
C1	I	1–3	0.14	0	100	0
		4–8	0.28	0	100	0
	II	9–15	0.47	25	75	1.0
		16–24	0.80	25	75	2.0
	III	25–56	1.20	25	75	2.0
	IV	57–76	0.70	25	75	1.0
C2	I	1–3	0.14	0	100	0
		4–8	0.28	0	100	0
	II	9–24	0.40	25	75	0
	III + IV	25–76	0.60	25	75	0
C3	I	1–3	0.12	0	100	0
		4–8	0.25	0	100	0
	II	9–15	0.45	25	75	1.0
		16–24	0.75	25	75	2.0
	III + IV	25–56	1.12	25	75	2.0
	V	57–94	0.50	25	75	0
	VI	95–132	0.67	25	75	1.0
C4	I	1–3	0.12	0	100	0
		4–8	0.25	0	100	0
	II	9–24	0.25	25	75	0
	III + IV + V	25–76^c^	0.50	25	75	0
	VI	95–132	0.50	25	75	0

a 25% *C. vulgaris* mixed with 75% PPW_dp_ or PPW_p_ had C/N ratios of 22.8 and 19.9, respectively.

b Digesters C1 and C2 were fed with potato discarded parts (PPW_dp_); digesters C3 and C4 were fed with potato peel (PPW_p_).

c During the recovery stage (from days 57 to 94), digester C4 was unfed for a period (days 77 to 94).

During the semi-contiguous co-digestion process, biogas yield, methane content, and pH values were measured every feeding day. Digestate samples were collected weekly and analysed for TS, VS, the concentrations of total chemical oxygen demand (COD_t_), soluble chemical oxygen demand (COD_s_), total alkalinity (TA), volatile fatty acids (VFAs), ammonium nitrogen (NH_4_^+^-N) and free ammonia nitrogen (FAN).

### 2.3 Analytical methods

The TS and VS of feedstocks and digester samples were determined according to APHA standard methods.^39^ Concentrations of COD_t_ and COD_s_ were measured using Merck Millipore COD cell test kits (VWR, UK) based on the standard methods of APHA 5220D.^39^ To obtain the soluble phase, samples were centrifuged at 3392 × *g* for 10 minutes and then filtered using a 0.2 μm nylon filter (VWR, UK). Feedstocks' carbohydrate content was measured *via* the phenol-sulfuric acid method, with d-glucose as a standard.^40^ Feedstocks' protein content was measured using a bicinchoninic acid (BCA) protein assay kit (Thermo Scientific Pierce, UK), using bovine serum albumin as the standard. For analysis of C and N content, feedstocks were oven dried at 60 °C until constant weight and analysed by using an Elementar VarioMAX CNS analyzer (Elementar, Germany). Concentration of NH_4_^+^-N of digester samples was measured using Merck ammonium cell test kits (VWR, UK) based on the standard methods of APHA 4500-NH_3_ F.^39^ pH was measured using a Jenway 3010 pH-meter (Jenway, UK), and FAN was calculated using eqn (1):^41^1
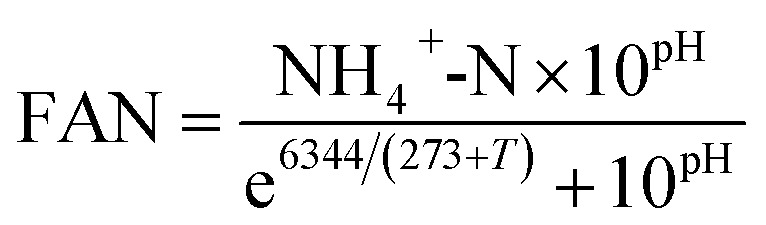


The total alkalinity (TA) and total VFAs concentrations were determined *via* titration with 0.1 N sulphuric acid as standard.^42^ The measurement of the individual concentrations of VFAs, samples were first diluted and then filtered using a 0.2 μm nylon filter (VWR, UK). Then, the filtered sample was mixed with 0.1 M octane sulphonic acid at a ratio of 1 : 1 before sonicating for 40 minutes. The VFAs were than measured by an Ion Chromatography (Dionex ICS-1000) equipped with an Ionpack ICE ASI column, with heptafluorobutyric acid as the eluent and tetrabutylammonium hydroxide as the regenerant. The IC also equipped with an AS-AP auto sampler and a Chameleon 7 Software to analyse the sonicated samples.

Gasbags (1 L) (Sigma-Aldrich, UK) were used for biogas collection from the digesters. On each measurement day, the gasbags were disconnected and biogas volume immediately quantified using a 500 mL jumbo gastight syringe (SGE, 500MAR-LL-GT). Methane content was analysed by gas chromatography. Subsequently, the gasbags were emptied and reconnected to the digesters. The methane composition were analysed by gas chromatography (Carlo-Erba 5160 GC with MFC 500 detector) in split mode with the injector at 150 °C and FID at 300 °C. The GC fitted with an Agilent HP-PLOTQ column (0.32 mm diameter, 30 m length and 20 μm film, Agilent, UK), and carrier gas was hydrogen (250 mL min^−1^) with an oven temperature held at 35 °C. At the beginning, triplicate injections of 50, 40, 30, 20 and 10 μL of methane standards (10% or 80% balanced with CO_2_; Scientific and Technical Gases Ltd., UK) to derive a standard curve. A minimum calibration coefficient (*R*^2^) of 0.99 is required before the analysis of test gas samples. After that, triplicate injections of a 50 μL of test sample, taken from the gas bags of the semi-continuous digesters using a 100 μL gastight syringe (SGE, 100R-V-GT), were qualified by reference to the standard curve. The volume of methane was calculated under STP conditions (0 °C, 1 atm).

### 2.4 Microbial analysis

For microbial community analysis, genomic DNA from samples of the semi-continuous digesters and the negative control were extracted using an isolation kit (DNeasy PowerSoil kit, QIAGEN, UK) following the manufacturer's instructions. After extraction, the quality of DNA samples were determined using a DeNovix spectrophotometer (DeNovix, US) measuring the absorbance at 260 and 280 nm. The extracted total DNA samples were sent for paired-end Illumina MiSeq sequencing of the V4 hypervariable region of 16S rRNA. The basic processes for Illumina MiSeq sequencing are library preparation, cluster amplification, sequencing and alignment and data analysis. The universal primers set 515F and 806R were used to allow amplification of the V4 region of both bacteria and archaea.^43^ The amplicon libraries were sequenced on the Illumina MiSeq platform using the Wet Lab SOP as described by Kozich *et al.* (2013).^44^ At the end, FASTQ files with quality score encoding were generated. The raw Illumina FASTQ files were demultiplexed and quality filtered using QIIME 2 with plugin wraps DADA 2 (https://docs.qiime2.org/2018.4/). Sequences presenting at 99% similarity were grouped into one operational taxonomic unit (OTU), and assigned taxonomy from the SILVA 119 reference database. The processed sequencing data were further analysed to check microbial diversity using the phyloseq package in RStudio.^45^

### 2.5 Statistical analysis

The independent Samples *t*-test and one-way analysis of variance (ANOVA) were utilized to test the effects of glycerol dose on the significance of methane production and digester performance by co-digestion of *C. vulgaris* and PPW using IBM SPSS statistics, version 23.^46^ Microbial alpha diversity of observed OTU numbers, Shannon's and Simpson's indices were plotted using phyloseq and ggplot2 packages in RStudio (version 3.5.3). Significant differences in alpha diversity between the operation time and glycerol addition were compared by means of ANOVA (*aov* function). Principal coordinate analysis (PCoA) was performed to examine the microbial beta diversity based on Bray–Curtis distance measures. Significant differences in beta diversity between the operation time and glycerol addition were identified by means of pair-wise permutational multivariate analysis of variance (PERMANOVA) with Bonferroni correction, using phyloseq and RVAideMemoire packages in RStudio (version 3.5.3). The independent Samples *t*-test was utilized to test the effect of glycerol dose on the significance of relative abundance of bacterial community, using IBM SPSS (version 23).^46^ A Spearman's rank-order correlation was run to determine the relationship between the relative abundance of methanogenic archaea and digester operating parameters, using IBM SPSS (version 23).^46^ A confidence interval of differences of 95% (*p* < 0.05) was chosen to define statistical significance.

## 3. Results and discussion

### 3.1 Effect of co-digesting *C. vulgaris* and PPW_dp_ with glycerol on AD performance

**Fig. 1:**
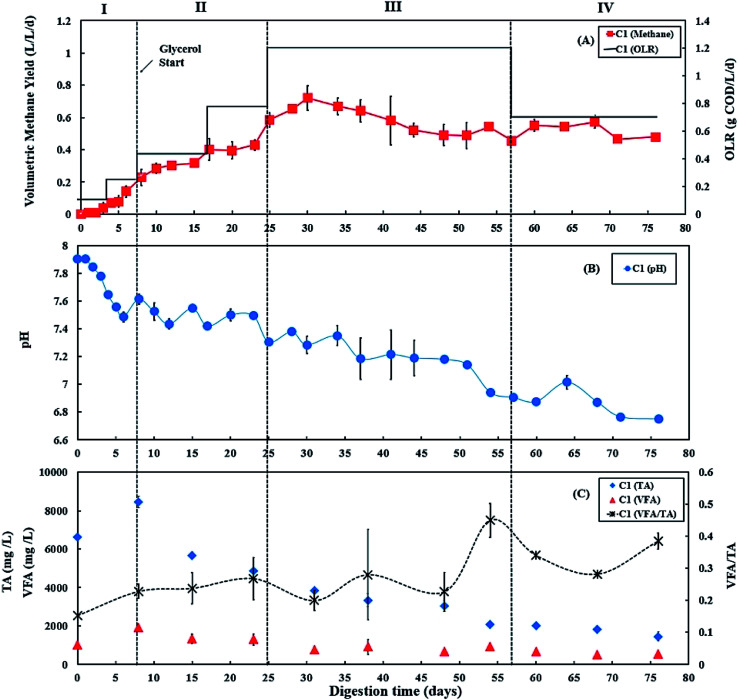
Variation in (A) volumetric methane yield, (B) pH, and (C) concentrations of total alkalinity (TA), volatile fatty acids (VFA) and ratio of VFA/TA during co-digestion of *C. vulgaris* and potato discarded parts (PPW_dp_) with glycerol (C1). Error bars = mean ± SD, *n* = 2.

**Fig. 2:**
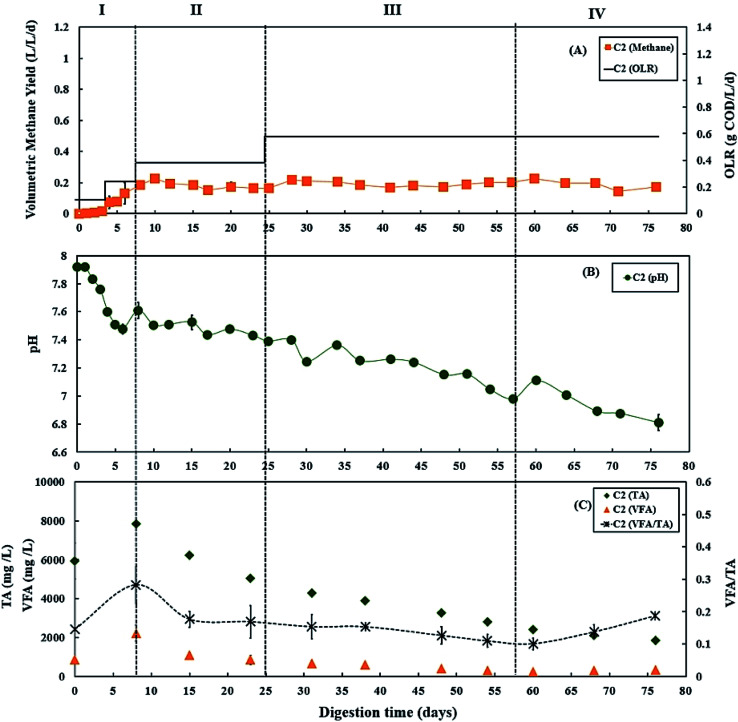
Variation in (A) volumetric methane yield, (B) pH, and (C) concentrations of total alkalinity (TA), volatile fatty acids (VFA) and ratio of VFA/TA during co-digestion of *C. vulgaris* and potato discarded parts (PPW_dp_) without glycerol (C2). Error bars = mean ± SD, *n* = 2.

During period III (days 25 to 56), the glycerol feed was maintained at 2% v/v with a high OLR at 1.20 g COD per L per day for digester C1 (Fig. 1A), whereas the OLR of digester C2 was kept at 0.60 g COD per L per day (Fig. 2A). The glycerol dosage significantly enhanced volumetric methane production (*F*(2,61) = 319.67, *p* < 0.001). Digester C1 (2% v/v dosage) had the mean methane production of 0.59 ± 0.08 L CH_4_ per L per d (Fig. 1 A), which was significantly higher than C2 (0.19 ± 0.02 L CH_4_ per L per d; *p* < 0.001) (Fig. 2A). Methane production of digester C1 rapidly increased with glycerol addition, likely due to catabolism of the readily biodegradable soluble COD in glycerol. This agrees with Wohlgemut *et al.* (2011) in which glycerol doubled methane production with a four times higher OLR when used as a co-substrate with pig manure.^32^

The maximum concentrations of TA and VFA were observed on day 8 for both digesters, after which they decreased continuously (Fig. 1C and 2C). During period III, from days 31 to 48, the average VFA/TA ratio of digester C1 was 0.24, whereas digester C2 had a lower ratio of 0.14. By day 54, VFA concentrations increased slightly to 929 ± 117 mg L^−1^ in digester C1 causing the VFA/TA ratio to peak at 0.45; Ciotola *et al.* (2014) and Pontoni *et al.* (2015) suggested that digesters are overloaded when this ratio exceeded 0.40.^47,48^ pH values for digester C1 also showed a declining trend (6.94 ± 0.01 by day 54) (Fig. 1B), although this remained within the optimum range of 6.8–7.2 for the AD process.^49^ These results agree with the work of Ciotola *et al.* (2014), who found that during AD of dairy manure with a high OLR (1.8 kg VS per m^3^ per day), the digester failed at a high VFA/TA ratio of 0.65, despite pH remaining at 6.92.^47^ This may have been due to the accumulation of short chain fatty acids leading to a significant reduction of buffering capacity before the pH dropped, as reported by Ward *et al.* (2008).^49^ Glycerol is rapidly consumed by acidogenic bacteria which generate large amounts of organic acids;^29,30^ therefore, balanced alkalinity is important for AD of glycerol.^31^ However, Astals *et al.* (2012) found that co-digesting pig manure with crude glycerol reduced the alkalinity because glycerol provides negligible alkalinity, resulting in the VFA/TA ratio exceeding 0.60.^33^ Therefore, in their study, the crude glycerol dosage was reduced from 5 to 4% w/w which decreased the VFA/TA ratio to less than 0.4 after two days. In the current study, Fig. 1C and 2C show that on day 54, digester C1 had a lower TA concentration (2062 ± 18 mg L^−1^) than digester C2 (2800 ± 35 mg L^−1^). Therefore, on day 57, the glycerol dosage was reduced to 1% v/v, consequently the OLR decreased to 0.70 g COD per L per day (Fig. 1A). Consequently, on day 60, the VFA/TA ratio of digester C1 decreased to 0.34, within the optimum range for stable AD. During period IV (days 57 to 76), the mean volumetric methane yield of C1 was 0.51 ± 0.05 L CH_4_ per L per d (Fig. 1A) being significantly higher than digester C2 (0.19 ± 0.03 L CH_4_ per L per d; *p* < 0.001) (Fig. 2A).

### 3.2 Effect of co-digesting *C. vulgaris* and PPW_p_ with glycerol on AD performance

**Fig. 3:**
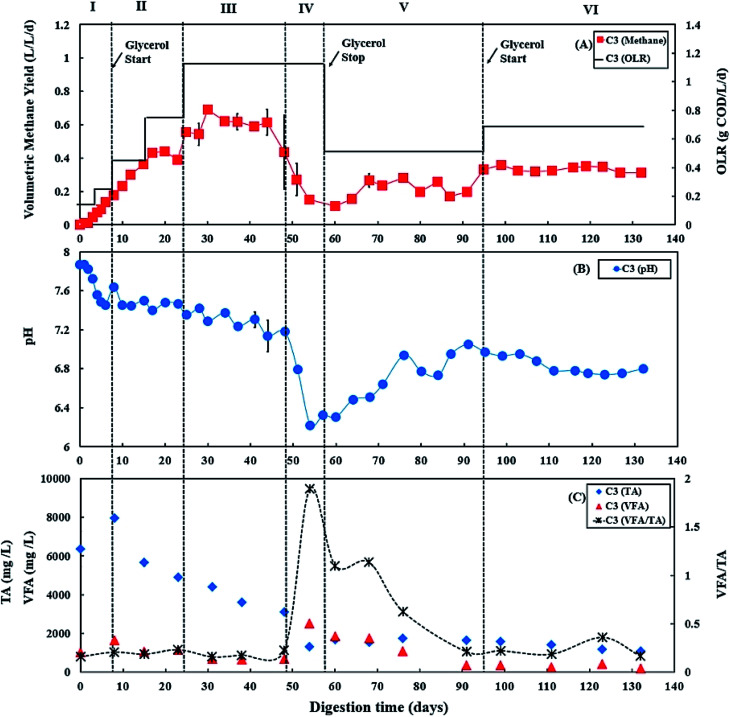
Variation in (A) volumetric methane yield, (B) pH, and (C) concentrations of total alkalinity (TA), volatile fatty acids (VFA) and ratio of VFA/TA during co-digestion of *C. vulgaris* and potato peel (PPW_p_) with glycerol (C3). Error bars = mean ± SD, *n* = 2.

**Fig. 4:**
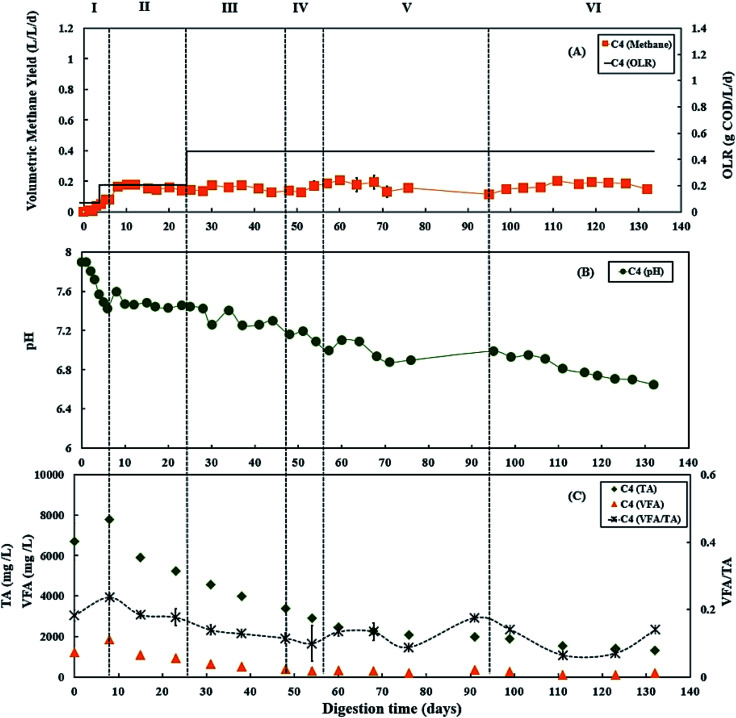
Variation in (A) volumetric methane yield, (B) pH, and (C) concentrations of total alkalinity (TA), volatile fatty acids (VFA) and ratio of VFA/TA during co-digestion of *C. vulgaris* and potato peel (PPW_p_) without glycerol (C4). Error bars = mean ± SD, *n* = 2.

During period III (from days 25 to 45), when the glycerol feed for digester C3 was increased to 2% v/v with a high OLR at 1.12 g COD per L per day. Digester C4 was maintained at 0.50 g COD per L per day of OLR. Fig. 3B and 4B show that there was a slow reduction of pH in both C3 and C4 during period III. The glycerol dosage significantly affected the volumetric methane production (*F*(2,65) = 916.41, *p* < 0.001). During period III, the mean volumetric methane production of 0.60 ± 0.05 L CH_4_ per L per d in digester C3, which was significantly higher than the level of 0.15 ± 0.02 L CH_4_ per L per d in digester C4 (*p* < 0.001).

The TA and VFA concentrations of digesters C3 and C4 also peaked after 8 days of operation, then decreased continuously until day 48 (Fig. 3C and 4C). Subsequently, the TA and VFA concentrations continued to decrease in digester C4, with a relatively stable VFA/TA ratio. However, digester C3 started to accumulate VFA and experienced reduced TA concentrations, resulting in the VFA/TA ratio peaking at 1.89 on day 54, corresponding with a significant drop in pH of digester C3 to 6.22 and decreased methane production. VFAs are crucial intermediate products affecting methane production and AD process stability, and glycerol degradation by acidogenic bacteria produces VFA. Propionic acid degrading microorganisms have lower specific growth rates than acetic acid- or butyric acid consumers that require longer degradation times.^50–52^ Therefore, Nielsen *et al.* (2007) and Xiao *et al.* (2015) suggested that propionic acid accumulation could be one of the major parameters indicating AD process instability.^53,54^ The growth rates of both acidogenic bacteria and methanogens are inhibited when propionic acid concentrations exceed 900 mg L^−1^, leading to reduced methane production.^55^ On day 54 of the current study, the total propionic acid concentration in digester C3 was 1220 mg L^−1^ (Fig. S2A in ESI†), which probably explains the reduction in methane yields in digester C3 at that time.

Lossie and Pütz (2008) suggested that the input of biomass to a digester should be reduced or stopped if the VFA/TA ratio exceeds 0.6.^42^ Therefore, on day 57, the feed of glycerol to digester C3 was stopped, with only the baseline feed of 25 : 75 *C. vulgaris* : PPW_p_ used to recover it during period V. The recovery stage of period V lasted for 37 days (from days 57 to 94), and the OLR of digester C3 was maintained at 0.50 g COD per L per day. During this recovery stage, pH, methane production and TA concentrations in digester C3 exhibited increasing trends (Fig. 3), while VFAs, especially propionic acid, decreased continuously, reducing the VFA/TA ratio from 1.89 to 0.21 (Fig. 3C). Lossie and Pütz (2008) also suggested that biomass loading should be increased slowly when the VFA/TA ratio ranges from 0.2 to 0.3.^42^ Therefore, after recovery, during period VI, glycerol feeding was restarted at 1% v/v to digester C3 on day 95, and the OLR was increased to 0.67 g COD per L per day (Fig. 3A). Period VI lasted 37 days (from days 95 to 132), the mean volumetric methane production of 0.33 ± 0.02 L CH_4_ per L per d in digester C3, which was significantly higher than 0.17 ± 0.03 L CH_4_ per L per d produced by digester C4 (*p* < 0.001).

### 3.3 Overall performance during co-digestion

The glycerol dosage level is a key factor affecting final methane production when using glycerol as a co-substrate because of its high COD concentration. Rapidly introducing high glycerol dosage would suddenly increase the OLR, and reduce digester performance by creating a “shock load” as described by Wohlgemut *et al.* (2011).^32^ In their study, the volumetric biogas/methane production stopped after 12 days because of VFA accumulation (>10 000 mg L^−1^) when pig manure was co-digested with 4% v/v glycerol. Similarly, co-digestion with 2% v/v glycerol also accumulated VFAs (>7000 mg L^−1^) after 25 days. During period II in the current study, a slow and stepwise increase in glycerol dosage from 1 to 2% v/v was implemented successfully without creating any organic shock load.

In the current study, the C/N ratios in the mixtures of 25% *C. vulgaris* with 75% PPW_dp_ or PPW_p_ were 22.8 and 19.9, respectively, both of which are within the optimum range for AD process.^18^ Co-digestion of *C. vulgaris* and PPW at this ratio could achieve stable digestion process as shown in Fig. 2 and 4. Although PPW_dp_ and PPW_p_ are both promising feedstocks for stable microalgae co-digestion, the addition of small amounts (1–2% v/v) of glycerol significantly enhanced volumetric methane yield (PPW_dp_: *F*(2,61) = 319.67, *p* < 0.001; PPW_p_: *F*(2,65) = 916.41, *p* < 0.001). The addition of glycerol also significantly enhanced specific methane yield (PPW_dp_: *F*(2,61) = 213.67, *p* < 0.001; PPW_p_: *F*(2,65) = 207.72, *p* < 0.001). Although the higher glycerol dose led to enhanced volumetric methane production, the highest specific methane yield was achieved with the lower dose. When mixtures of *C. vulgaris* : PPW_dp_ co-digesting with 1% v/v glycerol, the mean specific methane yield of 0.73 ± 0.07 L CH_4_ per g COD_added_ (based on total COD) was significantly higher than with 0% v/v dosage (mean of 0.30 ± 0.04 L CH_4_ per g COD_added_, *p* < 0.001) and 2% v/v dosage (mean of 0.49 ± 0.07 L CH_4_ per g COD_added_, *p* < 0.001). When mixtures of *C. vulgaris* : PPW_p_ co-digesting with 1% v/v glycerol, the specific methane yield (mean of 0.55 ± 0.03 L CH_4_ per g COD_added_) was significantly higher than with 0% v/v dosage (mean of 0.33 ± 0.05 L CH_4_ per g COD_added_, *p* < 0.001). The mean specific methane yield of 0.54 ± 0.04 L CH_4_ per g COD_added_ was observed when mixtures of *C. vulgaris* : PPW_p_ co-digesting with 2% v/v glycerol. There was no significant difference between the specific methane yield at the 2% and 1% v/v dosage (*p* = 0.687). Moreover, the addition of 2% v/v glycerol was more likely to accumulate VFA, resulting in high VFA/TA ratios, leading to a potentially unbalanced system. Consequently, 1% v/v glycerol appears to be the better dosage when applied to 25 : 75 co-digestion mixture of *C. vulgaris*/PPW. This agrees with Fountoulakis *et al.* (2010) and Panpong *et al.* (2014) who applied 1% v/v glycerol during co-digestion with sewage sludge or canned seafood wastewater, doubling the volumetric methane production and specific methane yield.^56,57^ However, their systems also showed signs of organic overloading because of increased VFA concentrations and decreased pH when the dosage exceeded 1% v/v.

In the current study, when adding glycerol to the mixtures of microalgae : PPW, the experimental specific methane yield exceeded the theoretical value (0.35 L CH_4_ per g COD under STP). Glycerol is a readily degradable substrate, and Nguyen (2014) found that digestion of glycerol achieved the specific methane yield of 0.75 L CH_4_ per g COD, which is higher than the theoretical value.^58^ Similar observations were reported by Fountoulakis *et al.* (2010) who investigated the effect of co-digesting glycerol and sewage sludge, finding that the addition of glycerol could supply the extra organic carbon that enhanced the growth of active biomass in terms of increased VS values, and consequently the observed methane production exceeded the theoretical yield.^56^ Ma *et al.* (2008) also demonstrated that the addition of glycerol to potato processing wastewater had a positive effect on the growth of the active biomass which increased the amount of VS of 3 g VS L^−1^ after glycerol addition.^59^ In the current study, at the end of period III, the VS values increased when adding 2% v/v glycerol (C1 and C3), which were significantly higher than the digesters (C2 and C4) without glycerol (*t*(6) = −7.799, *p* < 0.001) (Tables S1 and S2 in ESI†). Similarly, the VS values in digesters C1 and C3 were significantly higher than C2 and C4 at the end of period IV and period VI (*t*(6) = −3.243, *p* = 0.018) (Tables S1 and S2†). In the current study, the enhanced methane yield beyond theoretical values might be because the active biomass (increased VS values) was enhanced by adding glycerol. Apart from this, the potential biodegradable COD has been accumulated in the digester for some time could be another possible reason for the observed high specific methane yield. Specifically, in the current study, the seed inoculum was collected from a manure-based anaerobic digester, which normally requires high OLR (3 g COD per L per day) to degrade it.^60^ However, the low OLR used in the current study results in less biomass is required to degrade the daily feed, therefore the extra biomass in the inoculum would be degraded and produce extra methane production. Moreover, in the current study, the digesters were fed slowly with glycerol at low dosage during period II, and then were fed every two days. Consequently, the residual biomass in the digester may gradually adapt to the previously non-degradable material, which may start to degrade it and produce more methane. Similar observations were reported by Sayed *et al.* (1984), in their study, the observed percentage of COD conversion to methane higher than 100% (111–121%) when the slaughterhouse waste was fed into the anaerobic reactor slowly and intermittently.^61^ The current study has yielded important findings that glycerol as an additional feedstock could improve methane yields when co-digesting with microalgae and PPW. However, in order to minimise these influences, it suggests that the digesters should operate some time to deplete the internal source of methane. Therefore, validation of the result is required for the future study, such as prolong the start-up time of the anaerobic digester, to develop a clear understanding the performance of glycerol as co-substrate for future microalgae biorefineries.

FAN is regarded as the active component leading to ammonia inhibition in AD processes,^62^ and microalgae biomass is characterised by having high protein content which can lead to high ammonia concentrations and inhibition when used as a mono-digestion feedstock. In the current study, the FAN concentrations of all digesters were less than 10 mg L^−1^, lower than previously reported methanogenic toxicity levels of 80–150 mg L^−1^.^15,63^ Therefore, the current study demonstrated that co-digestion of *C. vulgaris* and PPW, both with and without glycerol, helps avoid the development of ammonia toxicity.

### 3.4 Microbial characteristics

#### 3.4.1 Comparison of community diversity and similarity

**Fig. 5:**
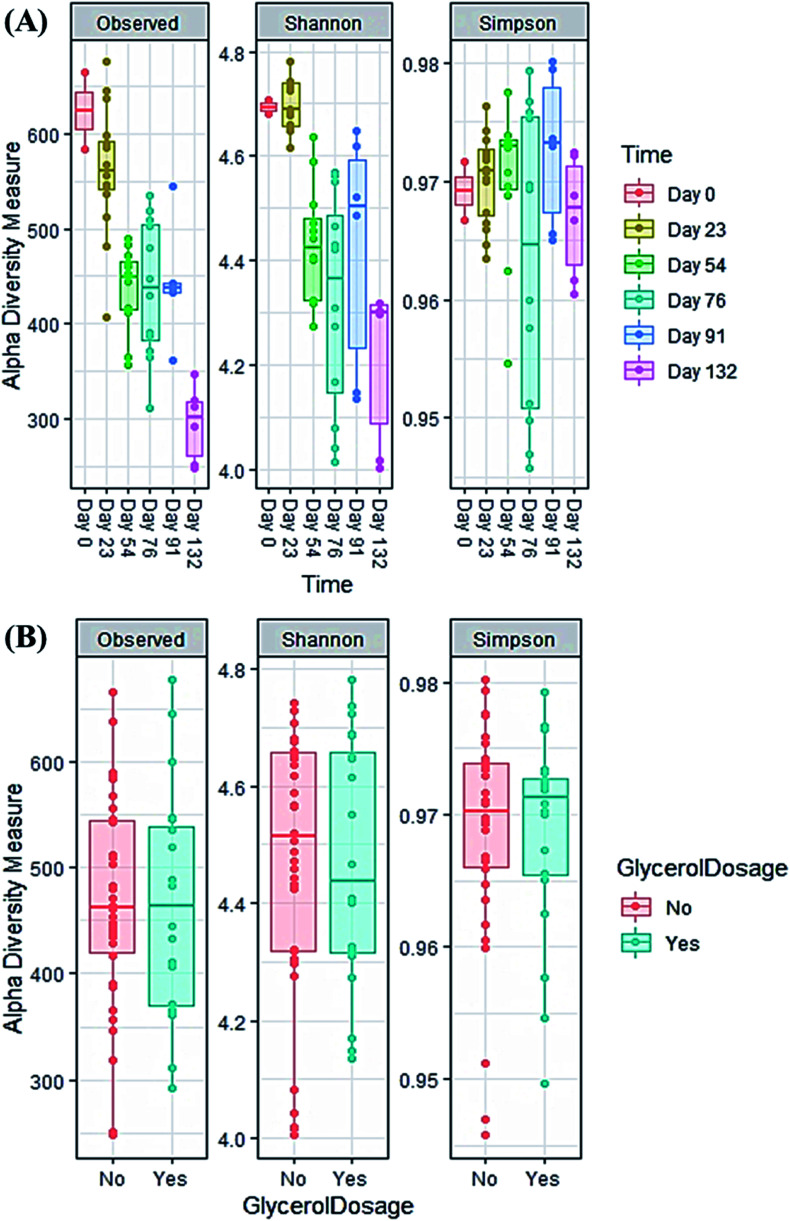
Alpha diversity indices of all samples during the co-digestion process: (A) time effect; (B) feedstock effect (with or without glycerol).

A comparison of α-diversity was used to determine differences in microbial community richness and evenness using observed OTU numbers, Shannon's and Simpson's indices.^64^ Operation time significantly reduced community richness (*p* < 0.001) (Fig. 5A). For example, OTU diversity was significantly higher in the inoculum (day 0) compared with day 54 (*p* = 0.002), day 76 (*p* = 0.002) and day 132 (*p* < 0.001). Operation time also significantly reduced Shannon diversity (*p* < 0.001) (Fig. 5A). The community on day 0 was more diverse than other sampling dates, but only significantly higher than day 76 (*p* = 0.02) and day 132 (*p* = 0.002). However, Simpson's index showed no statistical difference among the operation time (*p* = 0.097). Fig. 5A show that a decrease in alpha diversity was observed from days 0 to 76, and it increased again from days 76 to 91 (during the recovery phase). This agrees with De Vrieze *et al.* (2017) who found microbial alpha diversity was decreased during the disturbance period, but it increased again during the stabilisation period of the AD process.^65^Fig. 5B shows that there was no significant effect of glycerol addition on any of the alpha diversity measures.

**Fig. 6:**
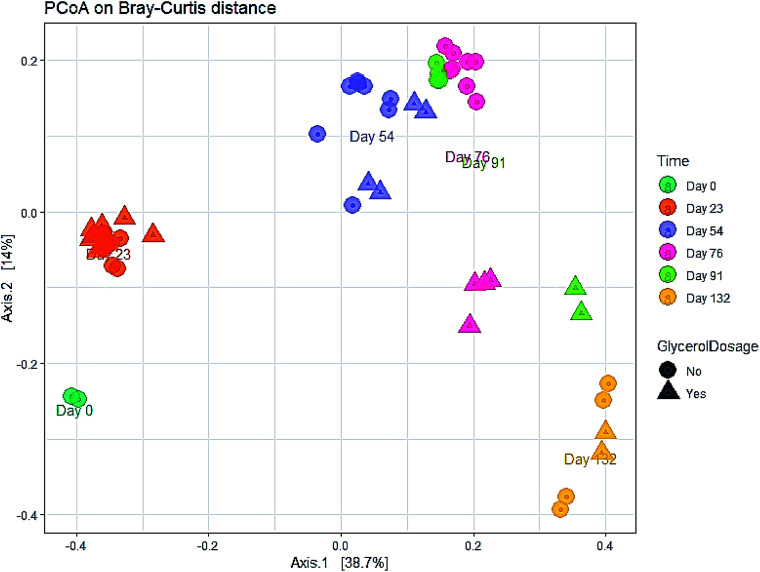
Principal coordinates plot (PCoA) of the microbial community during the co-digestion process based on Bray–Curtis distance matrix.

The evolution in microbial community dynamics (beta diversity) over the operation time and with glycerol addition was analysed *via* principal coordinate analysis (PCoA) of the Bray–Curtis distance measure (Fig. 6). Time had a significant effect on community development (*p* = 0.001), with distinct clustering identifiable for each sampling date; this is unsurprising given the need for the manure-adapted seed inoculum to respond to a markedly different feedstock employed in this study. Days 76 and 91 had substantial overlap (during the recovery phase), the community composition of these two sampling dates was not significantly different (*p* = 1.000). Moreover, the community composition between day 91 and day 132 was not significant (*p* = 0.06). Continued methane production under sub-optimal operating conditions highlights the functional resilience of the communities, even during the recovery phase.

A significant difference in community composition was also observed with glycerol addition (*p* = 0.024). Glycerol amended and unamended treatments grouped within distinct clusters, but with substantial overlap; likely due to switching glycerol feed rates in response to digester instability.

#### 3.4.2 Microbial community composition

**Fig. 7:**
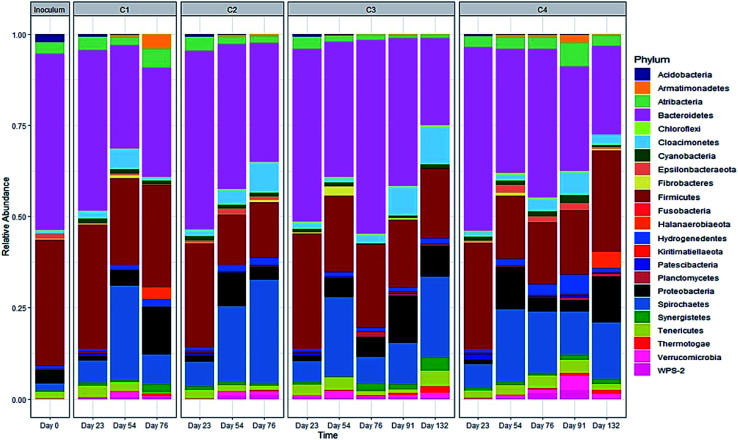
Relative abundance of the bacterial community at the phylum level (>2% of total sequence).

Fig. 7 shows the relative abundance of bacterial phyla in seed inoculum and other AD digesters. *Bacteroidetes* (48%) and *Firmicutes* (35%) were the two dominant phyla in the seed inoculum. The bacteria community was mainly dominated by *Bacteroidetes*, *Firmicutes* and *Spirochaetes* during the co-digestion process. *Bacteroidetes* abundance fluctuated somewhat in C3 and C4, but an overall decreasing trend in all treatments. *Firmicutes* abundance also exhibited a decreasing trend, at the end of digestion, a higher abundance of *Firmicutes* was observed in C1 and C4 (both at 28%). *Spirochaetes* abundance increased in all treatments from days 23 to 54, and then exhibited the decreasing trend in C1 and C4 while a slightly increased abundance was observed in C2 (from 21 to 28%). After the recovery stage, glycerol was back to add to digester C3 (from days 91 to 132), and *Spirochaetes* abundance showed an increasing trend (from 11 to 22%). In the current study, although the relative abundances of these three dominant phyla fluctuated somewhat in all AD conditions, the glycerol dosage had no significant effect on the relative abundances of *Bacteroidetes* (*p* = 0.902), *Firmicutes* (*p* = 0.348) and *Spirochaetes* (*p* = 0.649). Bacteria are responsible for the first three steps of AD process, and in comparison with methanogens, bacteria are normally not severely affected by the changes of operational conditions and/or the presentence of inhibitory substances, and consequently they may not pose severe problems.^66,67^

**Fig. 8:**
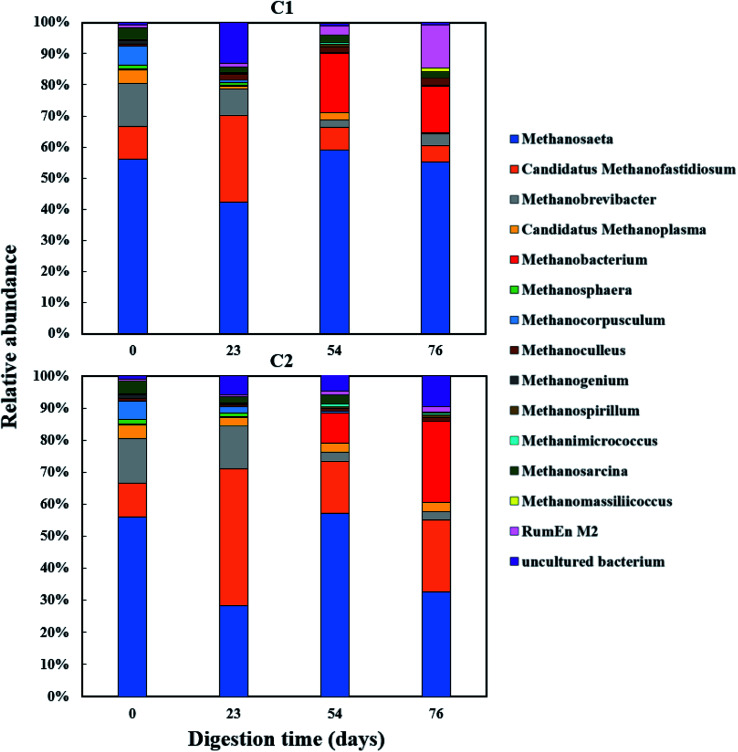
Relative abundance of methanogenic archaea (genus level) during co-digestion of *C. vulgaris* and potato discarded parts (PPW_dp_) with (C1) or without (C2) glycerol addition.

**Fig. 9:**
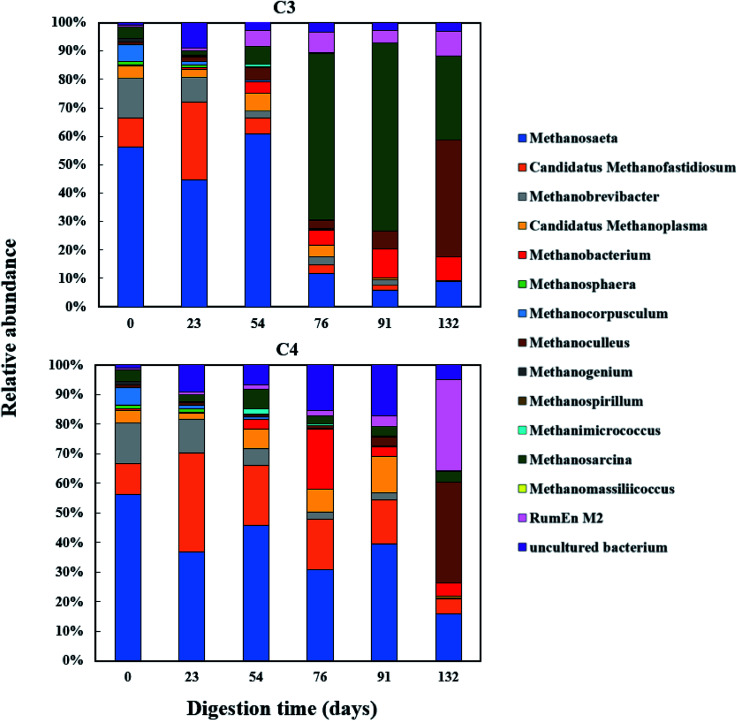
Relative abundance of methanogenic archaea (genus level) during co-digestion of *C. vulgaris* and potato peel (PPW_p_) with (C3) or without (C4) glycerol addition.

The relative abundance of the methanogens are presented in Fig. 8 and 9. Among the methanogens, *Methanosaeta* had the highest relative abundance in the seed inoculum and remained dominant up to day 54. *Methanosaeta* abundance fluctuated somewhat in C2 but an overall decreasing trend was evident over the extended sampling periods for digesters C3 and C4. *Methanosaeta*, as a specialist acetate degrader, is expected to be favored in low acetic acid environments (<100 mg L^−1^).^68,69^ It should be noted that the genus name *Methanosaeta* apply to the same taxon as *Methanothrix*.^70^ However, in the current study, to prevent confusion we use the established name (*Methanosaeta*) as it is generally used in very recent studies.^4,71^ From days 23 to 54, the total VFA concentrations of digesters C1, C2 and C4 decreased steadily, with acetic acid concentrations remaining below 100 mg L^−1^ (Fig. S1 and S2†); acetic acid concentration correlating negatively with *Methanosaeta* abundance (*r*_s_(34) = −0.395, *p* = 0.021). Digester C3 experienced performance inhibition by day 54 through VFA accumulation and low pH (Fig. 3A), and had to be recovered. During recovery, the high acetic acid concentration was effectively degraded, supporting a shift in dominance to *Methanosarcina* (up to 66% relative abundance). *Methanosarcina* is a robust acetoclastic methanogen that can utilize acetate, CO_2_, methyl-group containing compounds or H_2_ as substrate.^68^ It survives at pH 5–8 and is often associated with deteriorating digester performance.^69,72^ In C3, *Methanosarcina* abundance and methane production were negatively correlated (*r*_s_ (34) = −0.487, *p* = 0.004), with abundance exhibiting a decreasing trend from day 91 to 132. The relative abundance of *Methanoculleus* increased from 6 to 41% over this period, becoming the dominant genus in digester C3 at the end of the digestion process. Furthermore, *Methanosaeta* dominance was lost in digester C4 by day 132, yielding to *Methanoculleus* and the H_2_-dependent methylotroph *Methanomassiliicoccales* and the archaeon *RumEn M2* (Fig. 9).^73^*Methanoculleus* is a hydrogenotrophic methanogen that grows favourably at low acetate and hydrogen concentrations that were prevalent during the pseudo of period VI.^74^ De Vrieze *et al.* (2012) reported that a robust methanogenic process can be established based on syntrophic acetate oxidation coupled with hydrogenotrophic methanogenesis by *Methanosarcina* under elevated OLR conditions.^72^ Therefore, during period VI the high methane production achieved by digester C3 was probably a result of interactions between *Methanosarcina* and *Methanoculleus*. Other notable community features included the maintenance of high relative abundances of the methyl-reducing syntroph, *Candidatus Methanofastidiosum* (formerly WSA2)^75^ in digesters without glycerol (C2 and C4), and the strong growth of the hydrogentrophic methanogen *Methanobacterium*, which can utilize H_2_ (formate) to produce methane, in digesters C1 and C2 from day 54.

### 3.5 Technical and economic implications from co-digestion with glycerol

The current study shows glycerol to be a promising co-substrate for the co-digestion of microalgae and PPW to further enhance methane production. Previously, it has been discussed that the OLRs may potentially be increased when co-digesting microalgae with PPW.^22^ In the current study, glycerol supplementation allowed the operating OLRs to be further increased. Therefore, if the co-digestion of microalgae and PPW can be applied in large-scale AD, utilisation of glycerol as an additional feedstock may potentially further reduce the digester's size and capital costs.

Moreover, methane production was significantly enhanced when conducting co-digestion with glycerol at an optimum dosage of 1% v/v. When using microalgae as a substrate for biofuel production, one factor affecting financial viability is the transportation of the biomass to the operational site.^76^ In addition, feedstock availability is another important consideration when operating an AD plant. Therefore, when considering microalgae AD as part of a biorefinery concept, particularly where biodiesel production is one step in the value chain, glycerol will be produced on site thereby providing a readily available co-digestion feedstock and reducing the transportation costs. Another area which needs further consideration is the large amount of glycerol produced from biodiesel production that may require additional treatment, and this may potentially increase the cost of on-site waste management.^76^ Therefore, from an economic viewpoint, co-digestion with glycerol may be a technique that could be used to improve the overall efficiency of the AD process and the economics of the biorefinery plant. However, the utilisation of glycerol as a co-substrate requires a further economic viability analysis due to its high value as a chemical feedstock.^77^

To date, information concerning continuous digesters fed with microalgae biomass is still very limited compared to BMP tests. During the current work, the semi-continuous anaerobic tests focused on an evaluation of the potential benefits of using carbon-rich waste materials as co-digestion substrates. Since operating parameters such as OLR, HRT and mixing conditions are known to affect the performance of the AD process and influence methane production, it is therefore proposed that future work should investigate the effect of OLR and/or HRT as well as mixing conditions (*e.g.* continuous mixing) on the co-digestion of microalgae with potato processing waste and glycerol. The current study is focusing on studying the effect of glycerol dosage level on methane production when co-digestion with microalgae and PPW. The characteristics of glycerol may also have the potential effect on the co-digestion process, such as its C/N ratio and purity. Therefore, future research should be conducted to determine whether the C/N ratio and/or purity of glycerol have additional effect over the current study.

## 4. Conclusion

The feasibility of using glycerol as an additional co-substrate for the co-digestion of microalgae and PPW was evaluated in semi-continuous digester studies. When co-digesting with mixtures of *C. vulgaris* : PPW_dp_, the highest specific methane was achieved by 1% v/v glycerol dosage, which was significantly higher than 2% and 0% v/v dosage. When co-digesting with mixtures of *C. vulgaris* : PPW_p_, the highest specific methane yields was also achieved by 1% v/v glycerol dosage, which was significantly higher than 0% v/v dosage. However, there was no significant difference between 1 and 2% v/v dosage. Moreover, the 2% v/v dosage promoted the accumulation of VFA leading to an unstable process and requiring one treatment to be recovered. The microbial communities diverged markedly over operational time, and to a lesser extent in response to glycerol addition. The acetoclast *Methanosaeta* was abundant in all treatments but was replaced by *Methanosarcina* in the PPW_dp_ with glycerol treatment due to VFA accumulation. Overall, this study demonstrate that the performance of microalgae co-digestion is substantially improved by the addition of glycerol as an additional co-substrate, suggesting that 1% v/v could be the optimal dosage when co-digesting with mixtures of *C. vulgaris* : PPW to enhance methane production without organic overloading.

## Conflicts of interest

There are no conflicts to declare.

## Supplementary Material

RA-010-D0RA07840A-s001
