# Supplementary material for: Co-digestion of microalgae with potato processing waste and glycerol: effect of glycerol addition on methane production and the microbial community
Source: RSC Adv. 2020 Oct 9;10(61):37391–408. doi: 10.1039/d0ra07840a (PMC9057114; doi:10.1039/d0ra07840a)
Supplement: RA-010-D0RA07840A-s001 [file RA-010-D0RA07840A-s001.pdf]

Electronic Supplementary Material (ESI) for RSC Advances.
This journal is © The Royal Society of Chemistry 2020

Supplementary Information

Co-digestion of microalgae with potato processing waste and glycerol: effect of glycerol addition on methane production and the microbial community

Yanghanzi Zhang[a] *, Gary S. Caldwell[b], Philip T. Blythe[a], Andrew M. Zealand[c], Shuo Li[a], Simon Edwards[a], Jin Xing[a], Paul Goodman[a], Paul Whitworth[b], Paul J. Sallis[a]

[a] School of Engineering, Newcastle University, Cassie Building, Claremont Road, Newcastle upon Tyne, NE1 7RU, UK

[b] School of Natural and Environmental Sciences, Newcastle University, Ridley Building, Claremont Road, Newcastle upon Tyne, NE1 7RU, UK

[c] Department of Applied Sciences, Faculty of Health and Life Sciences, Northumbria University, Newcastle Upon Tyne NE1 8ST, UK

*corresponding author: yanghanzi.zhang@newcastle.ac.uk

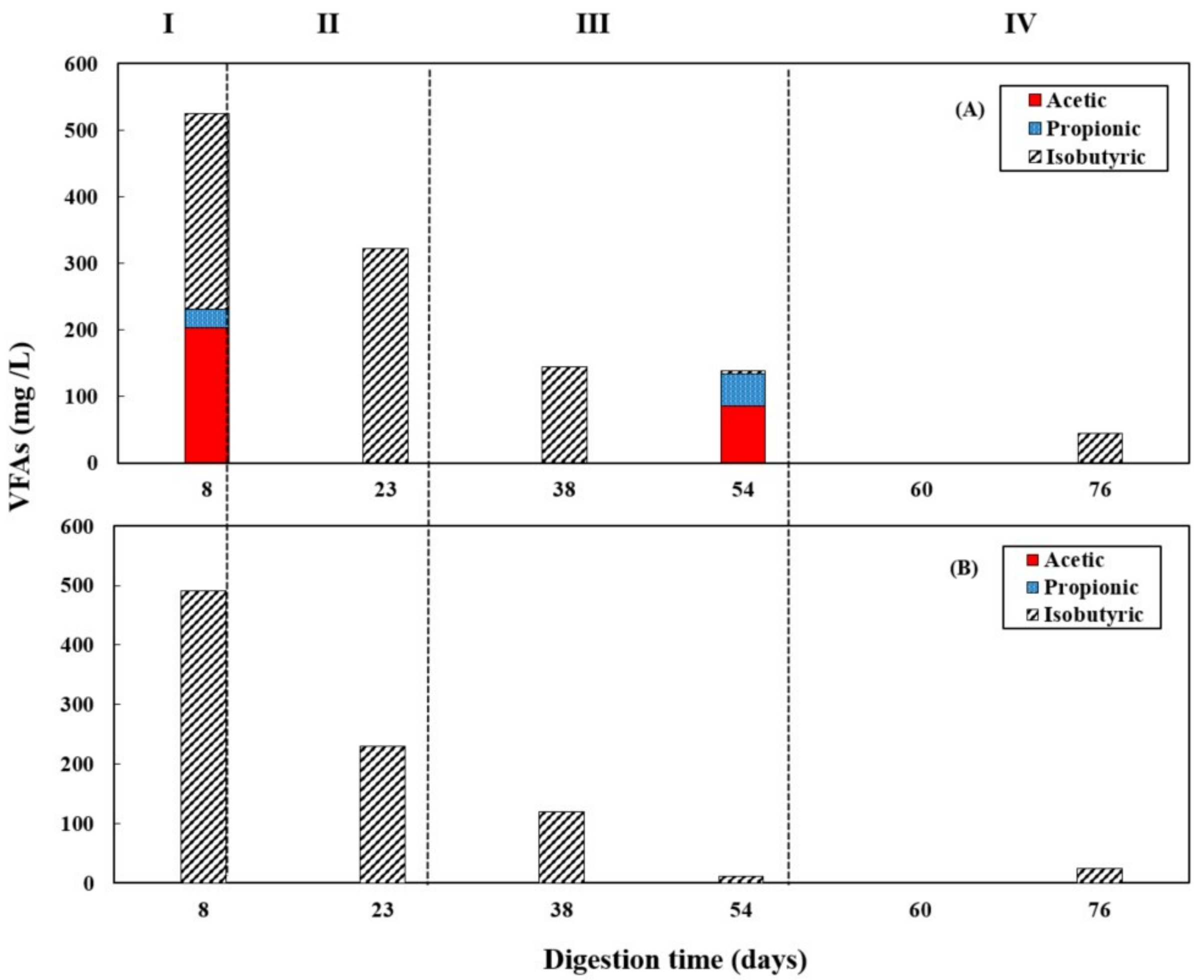

Figure S1. Concentrations of acetic, propionic and isobutyric acids in semi-continuous anaerobic co-digestion of *C. vulgaris* and potato discarded parts (PPW_{dp}) (A) with glycerol (C1) and (B) without glycerol (C2). Note: acetic and proponic acids below detection in all digester C2, and some C1 periods.

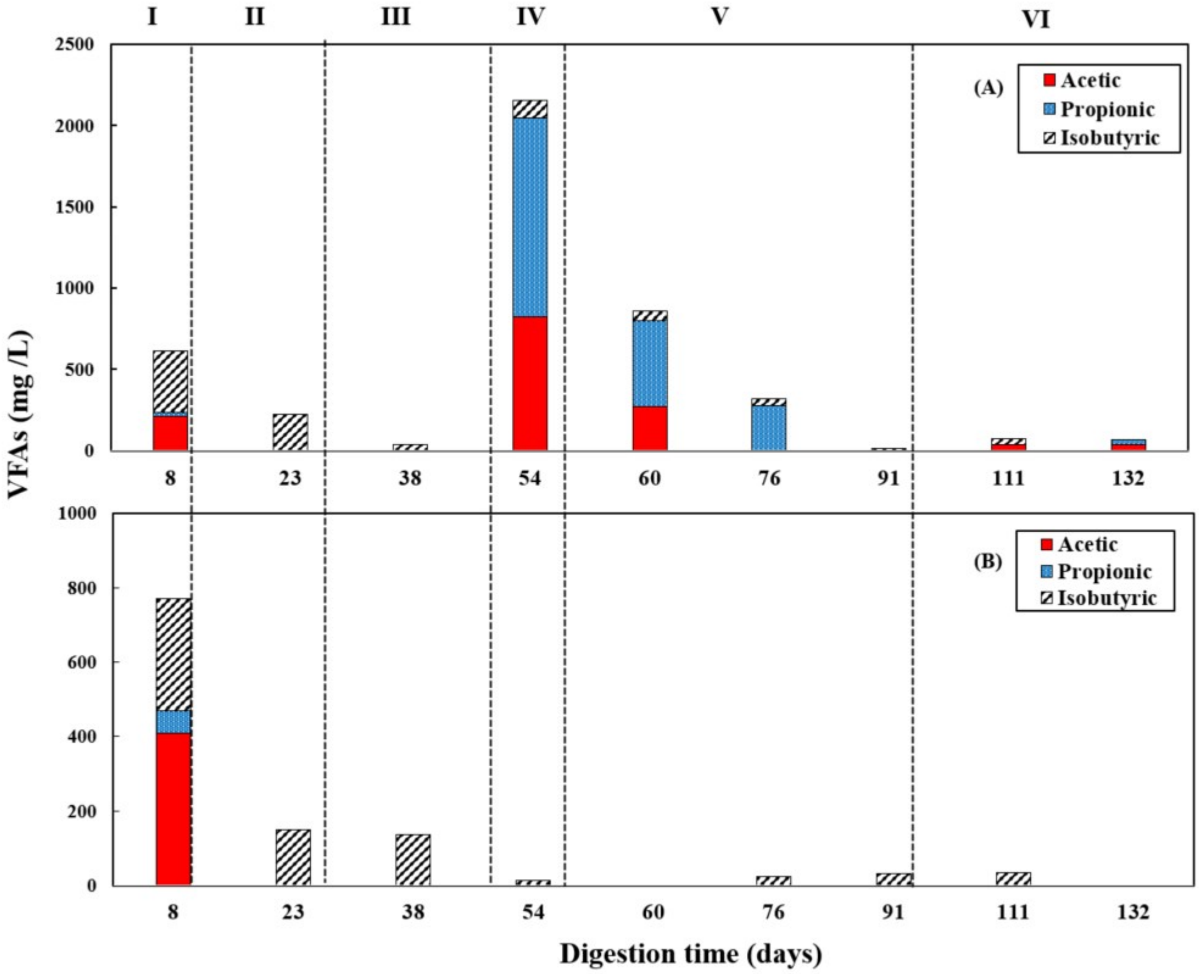

Figure S2. Concentrations of acetic, propionic and isobutyric acids in semi-continuous anaerobic co-digestion of *C. vulgaris* and potato peel (PPW_p) (A) with glycerol, digester C3 and (B) without glycerol, digester C4. Note: acetic and proponic acids below detection in some periods of digesters C3 and C4.

Table S1. Performance of digesters co-digesting *C. vulgaris* and PPW_{dp} with or without glycerol during different experimental stages.

	**Digester C1**		**Digester C2**	
	**Period III (day 24-56)**	**Period IV (day 57-76)**	**Period III (day 24-56)**	**Period IV (day 57-76)**
**OLR (g COD/L/d)**	1.20	0.70	0.60	0.60
**Process stability**				
pH	6.94 ± 0.01[a]	6.75 ± 0.01	7.05 ± 0.01	6.81 ± 0.06
Total Alkalinity (g/L)	2.06 ± 0.02	1.45 ± 0.25	2.80 ± 0.04	1.86 ± 0.02
Total VFAs (g/L)	0.93 ± 0.11	0.56 ± 0.06	0.31 ± 0.06	0.35 ± 0.01
NH^{+}_{4}-N (g/L)	0.34 ± 0.01	0.32 ± 0.01	0.53 ± 0.02	0.35 ± 0.04
FAN (mg/L)	3.86 ± 0.21	2.35 ± 0.21	7.56 ± 0.60	2.86 ± 0.73
**Digester effluent**				
TS (g/L)	8.09 ± 0.19	5.67 ± 0.01	6.79 ± 0.35	4.70 ± 0.39
VS (g/L)	5.36 ± 0.05	3.81 ± 0.01	4.18 ± 0.32	2.77 ± 0.37
COD_t (g/L)	6.86 ± 0.06	6.04 ± 0.06	5.84 ± 0.01	4.43 ± 0.08
COD_s (g/L)	1.49 ± 0.02	1.01 ± 0.05	1.25 ± 0.04	0.72 ± 0.06
$VS_{removal}$ (%)	49 ± 7	64 ± 5	57 ± 1	71 ± 1
$COD_{removal}$ (%)	59 ± 7	64 ± 6	69 ± 2	77 ± 2
**Methane production**				
Volumetric CH_4 yield (L/L/d)	0.59 ± 0.08	0.51 ± 0.05	0.19 ± 0.02	0.19 ± 0.03
Specific CH_4 yield (L/g COD_{added})	0.49 ± 0.07	0.73 ± 0.07	0.32 ± 0.03	0.32 ± 0.05

[a] Mean ± SD, n = 2.

Table S2. Performance of digesters co-digesting *C. vulgaris* and PPW_p with or without glycerol during the different experimental stages.

	**Digester C3**		**Digester C4**	
	**Period III (day 24-45)**	**Period VI (day 95-132)**	**Period III (day 24-45)**	**Period VI (day 95-132)**
**OLR (g COD/L/d)**	1.12	0.60	0.50	0.50
**Process stability**				
pH	7.18 ± 0.01[a]	6.80 ± 0.01	7.16 ± 0.01	6.65 ± 0.01
Total Alkalinity (g/L)	3.10 ± 0.14	1.10 ± 0.01	3.39 ± 0.02	1.30 ± 0.01
Total VFAs (g/L)	0.70 ± 0.02	0.18 ± 0.01	0.39 ± 0.06	0.18 ± 0.01
NH^+_4-N (g/L)	0.53 ± 0.01	0.16 ± 0.01	0.57 ± 0.02	0.18 ± 0.04
FAN (mg/L)	10.4 ± 0.23	1.28 ± 0.03	10.5 ± 0.16	1.07 ± 0.04
**Digester effluent**				
TS (g/L)	8.75 ± 0.05	4.51 ± 0.01	7.22 ± 0.03	4.02 ± 0.01
VS (g/L)	5.70 ± 0.15	3.30 ± 0.01	4.34 ± 0.09	2.85 ± 0.01
COD_t (g/L)	10.7 ± 0.43	4.88 ± 0.01	7.67 ± 0.42	4.10 ± 0.01
COD_s (g/L)	3.02 ± 1.91	0.88 ± 0.01	1.07 ± 0.01	0.41 ± 0.02
$VS_{removal}$ (%)	48 ± 6	70 ± 3	56 ± 1	71 ± 1
$COD_{removal}$ (%)	50 ± 5	77 ± 1	58 ± 5	78 ± 2
**Methane production**				
Volumetric CH_4 yield (L/L/d)	0.60 ± 0.05	0.33 ± 0.02	0.15 ± 0.02	0.17 ± 0.03
Specific CH_4 yield (L/g COD_{added})	0.54 ± 0.04	0.55 ± 0.03	0.31 ± 0.04	0.34 ± 0.06

[a] Mean ± SD, n = 2.
